# Regulatory landscape of AGE-RAGE-oxidative stress axis and its modulation by PPARγ activation in high fructose diet-induced metabolic syndrome

**DOI:** 10.1186/s12986-016-0149-z

**Published:** 2017-01-13

**Authors:** Luca Cannizzaro, Giuseppe Rossoni, Federica Savi, Alessandra Altomare, Cristina Marinello, Thammakorn Saethang, Marina Carini, D. Michael Payne, Trairak Pisitkun, Giancarlo Aldini, Asada Leelahavanichkul

**Affiliations:** 1 Systems Biology Center, Faculty of Medicine, Chulalongkorn University, Bangkok, 10330 Thailand; 2 Department of Pharmaceutical Sciences, Università degli Studi di Milano, Via Mangiagalli 25, 20133 Milan, Italy; 3 Department of Medical Biotechnology and Translational Medicine, Università degli Studi di Milano, Via Vanvitelli 32, 20129 Milan, Italy; 4 Pathological Anatomy Unit (U.O.C. Anatomia Patologica), ASST Santi Paolo e Carlo, Via di Rudinì 8, 20142 Milan, Italy; 5 Center of Excellence in Immunology and Immune-mediated Diseases, Department of Microbiology, Faculty of Medicine, Chulalongkorn University, Bangkok, 10330 Thailand

**Keywords:** AGE-RAGE-oxidative stress axis, Fructose, HNE, Metabolic syndrome, PPARγ, Rosiglitazone

## Abstract

**Background:**

The AGE-RAGE-oxidative stress (AROS) axis is involved in the onset and progression of metabolic syndrome induced by a high-fructose diet (HFD). PPARγ activation is known to modulate metabolic syndrome; however a systems-level investigation looking at the protective effects of PPARγ activation as related to the AROS axis has not been performed.

The aim of this work is to simultaneously characterize multiple molecular parameters within the AROS axis, using samples taken from different body fluids and tissues of a rat model of HFD-induced metabolic syndrome, in the presence or absence of a PPARγ agonist, Rosiglitazone (RGZ).

**Methods:**

Rats were fed with 60% HFD for the first half of the treatment duration (21 days) then continued with either HFD alone or HFD plus RGZ for the second half.

**Results:**

Rats receiving HFD alone showed metabolic syndrome manifestations including hypertension, dyslipidemia, increased glucose levels and insulin resistance, as well as abnormal kidney and inflammatory parameters. Systolic blood pressure, plasma triglyceride and glucose levels, plasma creatinine, and albuminuria were significantly improved in the presence of RGZ. The following molecular parameters of the AROS axis were significantly upregulated in our rat model: carboxymethyl lysine (CML) in urine and liver; carboxyethyl lysine (CEL) in urine; advanced glycation end products (AGEs) in plasma; receptor for advanced glycation end products (RAGE) in liver and kidney; advanced oxidation protein products (AOPP) in plasma; and 4-hydroxynonenal (HNE) in plasma, liver, and kidney. Conversely, with RGZ administration, the upregulation of AOPP and AGEs in plasma, CML and CEL in urine, RAGE in liver as well as HNE in plasma and liver was significantly counteracted/prevented.

**Conclusions:**

Our data demonstrate (i) the systems-level regulatory landscape of HFD-induced metabolic syndrome involving multiple molecular parameters, including HNE, AGEs and their receptor RAGE, and (ii) attenuation of metabolic syndrome by PPARγ modulation.

**Electronic supplementary material:**

The online version of this article (doi:10.1186/s12986-016-0149-z) contains supplementary material, which is available to authorized users.

## Background

Chronic non-communicable diseases such as diabetes, heart disease and cancer are responsible for 35 million deaths annually [1]. Many of these diseases are frequently associated with a complex disorder called metabolic syndrome, which is characterized by a group of co-occurring conditions (though not necessarily all occurring in the same patient): abdominal obesity, increased blood pressure, increased glucose level and multiple dyslipidaemias [2]; insulin resistance is also frequently found to occur in a subset of patients exibiting this syndrome [3]. These factors are typically associated with an increased risk of developing cardiovascular disease, type 2 diabetes mellitus and renal disease [1, 3, 4]. Both the underlying pathogenic mechanisms, as well as specific diagnostic criteria for metabolic syndrome remain incompletely defined, but this syndrome is known to be strongly related to consumption of high levels of fructose [1, 3, 4]. Most of the studies on the pathogenesis of metabolic syndrome have focused on advanced stages of the disorder. Recently, non-enzymatic protein glycation and lipoxidation have emerged as major candidates for initiating and sustaining this syndrome [4].

Fructose and the corresponding metabolic oxidation by-products such as methylglyoxal (MGO) react non-enzymatically with nucleophilic substrates such as proteins, forming adducts called advanced glycation end-products (AGEs) [5]. Among the numerous AGEs produced in vivo, two specific products, carboxymethyl lysine (CML) [6] and carboxyethyl lysine (CEL) [7], are especially abundant. AGEs interact with their specific cell-surface receptor, RAGE, initiating a signalling cascade that activates the transcription factor nuclear factor-kappa B (NF-κB), leading to increased release of inflammatory cytokines such as TNF-α [8]. The resulting increase in inflammation contributes to cellular dysfunction and tissue destruction. Moreover AGEs down-regulate intracellular detoxifying mechanisms. The result is the intensification of conditions such as diabetes [8].

This AGE-RAGE axis, activating NADPH oxidases and/or by other analogous mechanisms, also induces the formation of reactive oxygen species (ROS), which further increase cellular oxidative damage [8]. Advanced lipoxidation end-products (ALEs), including 4-hydroxynonenal (HNE), an α,β-unsaturated hydroxyalkenal, are formed by a mechanism involving lipid peroxidation; these reactive species can subsequently form adducts with proteins, often resulting in protein dysfunction. Accumulating evidence shows that a high-fructose diet (HFD) can also increase HNE production through activation of the AGE-RAGE-oxidative stress (AROS) axis [9–13]. Despite the increasing number of studies, the exact role of the AROS axis in the pathogenesis and progression of metabolic syndrome is still not completely understood. Importantly, these previous studies also have not evaluated all of the relevant parameters affecting production of AGEs and ALEs simutanuously, within a single model system.

A promising approach for both studying and treating metabolic syndrome involves the use of a class of drugs called thiazolidinediones [14–17], previously used to treat diabetes. Rosiglitazone (RGZ), the prototypical drug in this class, activates the nuclear peroxisome proliferator-activated receptor γ (PPARγ), a type II nuclear receptor, which in turn alters the expression of numerous genes [18], ultimately resulting in reduced blood concentrations of glucose, fatty acids, and insulin [19]. RGZ has also been shown to reduce the accumulation of AGEs [20–25], but little is known about the possible influence of RGZ on levels of the lipid peroxidation product, HNE.

Although there is no single known effective drug treatment for all the components of metabolic syndrome [26], there is a strong interest in developing (i) new molecules that maintain and extend RGZ therapeutic effects without its toxic side effects, and (ii) drugs specifically targeting the PPARγ receptor to benefit multiple diseases or co-occurring conditions in metabolic syndrome [27–30]. The specific aim of this study was to apply a systems biology approach for comprehensive evaluation of the multiple molecular modifications taking place via stimulation of the AROS axis, both without and with agonist potentiation of PPARγ activity by RGZ. The results provide a more complete picture of the landscape of the AROS axis, and illustrate the potential value of a systems approach in evaluating new drug candidates and the complex molecular mechanisms involved in metabolic syndrome.

## Methods

### Diet

The control diet contained 60% corn starch (carbohydrates), 20% casein (protein), 0.3% methionine, 5% lard (fat), 8% cellulose, 5% mineral mixture, 1% vitamin mixture, and zinc carbonate 0.004%. The fructose diet contained all the ingredients except corn starch, which was replaced by an equal quantity of fructose. Both diets were in pellet form. The diets were supplied by Diete Speciali, Mucedola s.r.l., Italy.

### Animals

Fifteen male Sprague-Dawley rats (Harlan Laboratories Inc., San Pietro al Natisone, Udine, Italy) weighing 200 ± 20 g (8 weeks of age) were studied. Rats were housed under constant environmental conditions (22 ± 1 °C, 50 ± 5% relative humidity, 12-h light/12-h dark cycle), with standard laboratory rat chow (batch 221644) or 60% high fructose diet (batch 221643) obtained from Mucedola s.r.l., Settimo Milanese, Milan, Italy and tap water ad libitum. Animals were acclimatized for a period of at least seven days before the use. The study was approved (protocol n° 16/2010) by the Animal Ethics Committee of University of Milan, Italy and communicated to the Italian Ministry of Health, having regard to the article seven of the D.L. 116/92. In addition, the study was carried out in strict accordance with the recommendations in the Guide for the Care and Use of Laboratory Animals published by the US National Institutes of Health (NIH Publication No. 85–23, revised 1996). All efforts were made to minimize animal suffering.

### Urine, plasma and tissue sampling

At the end of the experiment, rats within each group were placed in individual metabolic cages (#3600 M021; Tecniplast S.p.A, Buguggiate, Varese, Italy) for urine collections while continuing to have access to food and water. After a 24 h acclimation period, urine samples were collected for a 24 h period, and then the animals were returned to their usual cages. During the 24 h period spent in the metabolic cage, the animals did not receive RGZ. One ml of a 360 mM BHT ethanolic solution was added to the urine conical collection tubes (maintained at 4 °C), and 5 ml aliquots were frozen and stored at -80 °C until analysis.

At the end of the experiment, blood samples were collected from the tail vein in heparinized capillary tubes. Rats were anaesthetized with 60 mg/kg i.p. thiopentone sodium (Abbott s.r.l, Roma, Italy) and blood was collected from the inferior cava vein. Blood samples were centrifuged for 15 min. (2000 g at 4 °C), and plasma samples (500 μl aliquots) stored at -80 °C until analysis. The animals were then killed by decapitation for kidney and liver harvesting. The harvested organs were immediately frozen in liquid nitrogen, and stored at -80 °C until assayed. Just before analysis, tissue samples were thawed, rinsed in ice-cold physiological saline, weighed and minced [31].

### Indirect systolic blood pressure (SBP) and heart rate measurements

At the end of the experiment, SBP was measured in conscious rats by tail-cuff plethysmography [32]. The animals where pre-warmed at 37 °C for 30 min., and the measurements were obtained with the rats restrained in a plastic chamber without anaesthesia. A pneumatic pulse transducer positioned on the ventral surface of the tail distal to the occlusion cuff detected the return of the pulse after a slow deflation of the cuff. Cuff pressure was determined by a pneumatic pulse transducer with a programmed electro-sphygmomanometer (mod 58500; Ugo Basile, Comerio, Varese, Italy). SBP values for individual rats were obtained from the average of three consecutive measurements and were considered valid only when these readings did not differ by more than 5 mmHg. At the same time, heart rate was measured from the arterial pulse wave.

### Experimental design

At the beginning of our study, rats were randomly divided into three groups. One group was maintained on standard rat chow diet for six weeks (CTR group), whereas the other two groups were given fructose-enriched diet for six weeks. For the two groups that received fructose-enriched diet, one group was also given Rosiglitazone (10 mg ⁄ kg ⁄ day, in powder form; Glaxo-Smith-Klein, Middlesex, UK) three weeks after the initiation of the diet (RGZ group) and the other one was continued with fructose-enriched diet alone (HFD group). RGZ was orally administered by gastric gavage to rats during the last three weeks of the study. The compound were dissolved in a 5% (v/v) DMSO/H_2_O solution and diluted with 1% PEG400 (v/v) distilled H_2_O solution and kept protected from light throughout the study. During all the experiments, rats had free access to water and food. The rats were placed in metabolic cages (Tecniplast S.p.A., Buguggiate, Italy).

### Blood and urine biochemistry

Plasma TNF-α and IL-6 were determined by ELISA using a commercially available kit (R&D Systems, Inc., USA) according to the manufacturer’s instructions.

Advanced oxidation protein products (AOPP) were quantified according to the method previously described by Witko-Sarsat et al.[33].

Plasma and urinary fluorescent AGEs were determined by using a spectrofluorimeter (Perkin Elmer LS50B) setting the excitation wavelength at 370 nm and emission at 440 nm. AGE values were expressed as fluorescence units (FU)/ml for plasma samples and in 24 h for urines.

Plasma and urine carboxymethyl lysine (CML), carboxyethyl lysine (CEL) levels were measured by the use of commercially available kit: ELISA kit (OxiSelect™ CML ELISA Kit, Cell Biolabs Inc., OxiSelect™ CEL ELISA Kit, Cell Biolabs Inc.). Plasma and urine were diluted and tested in duplicate. The assay was performed according to the manufacturer’s instructions. Absorbance was read on a microplate reader: Wallac Victor 2 Wallac 1420 workstation.

### Surrogate indices of steatosis and/or parameters of impaired fat metabolism

Aspartate aminotransferase (AST), alanine transaminase (ALT), hepatic total lipids, hepatic triglycerides and hepatic total cholesterol were measured as previously described [34].

### Preparation of lysate from tissues

Tissues were dissected with clean tools, on ice, as quickly as possible to prevent degradation by proteases. The tissues pieces were placed in round-glass tubes. Lysis buffer (20 mM Tris-HCl, pH 7.5, 0.1% SDS, 1% Triton X-100, 1 mM Na_2_EDTA, 1% Protease inhibitors cocktail) without beta-mercaptoethanol was added in proportion 1: 2 (w/v) to the small pieces of tissues and put on ice. Homogenization was performed using an Ultra Turrax T25 (Janke & Kunkel, IKA Labortechnik). 2-Mercaptoethanol was added, samples were sonicated and centrifuged for 20 min at 13,000 g at 4 °C in a microcentrifuge MIKRO 120 HETTICH zentrifugen). The Triton X-100-soluble supernatant fraction (Sup) was removed, the Bradford protein assay was performed and the samples were stored at -80 °C.

Laemmli buffer (4X) was added to the Triton X-100-insoluble fraction (Pellet) (which enriches for detergent-resistant membranes, such as lipid rafts) at the bottom of the tubes (1:1, v/v), incubated 20 min at 95 °C, and centrifuged at 13,000 g, 5 min. The protein assay was then performed on the solublized Pellet fraction before storing the samples at -80 °C.

### Preparation of samples and SDS-PAGE

For Sup fractions, Laemmli Buffer 2X with beta-mercaptoethanol was added to samples 1:1(v/v), and heated for 5 min in Thermobloc FALC. One part of the sample was further diluted with two parts of Laemmli buffer with beta-mercaptoethanol and centrifuged 3 min. 20 μg of proteins were loaded on Any kD™ Mini-PROTEAN® TGX™ Precast Protein Gels (Bio-Rad) for SDS-PAGE. For Pellet fractions, each sample was heated for 5 min, taken one part and added to nine parts of Laemmli Buffer and centrifuged 3 min. 20 μg of proteins were loaded for SDS-PAGE. Precision Plus Protein™ WesternC Standards, Bio-Safe Coomassie Stain, 10x Tris/Glycine/SDS, Bio-Safe Coomassie Stain purchased from Bio-Rad were used to perform SDS PAGE.

Spectrophotometric measurements for protein assay were carried out with a computer-aided PerkinElmer UV/Vis Lambda 16 spectrophotometer.

### Detection and densitometric quantification of CML, HNE, RAGE content

Rabbit Anti-CML Antibody and Rabbit Anti-HNE polyclonal antibody were obtained from Cell Biolabs, Anti-beta actin Goat Anti-Mouse IgG, HRP-conjugate from Ambion (Life Technologies), Goat anti-RAGE (N-16) polyclonal antibody from Santa Cruz Biotechnology (Santa Cruz, CA). The homogenates were resolved by SDS-PAGE followed by electrophoretic gel transfer to Trans-Blot Transfer Pack, 0.2 μm nitrocellulose membrane (Bio-Rad) with a Trans Blot Turbo (Bio-Rad). Electrotransfer was carried out at 25 limit V, 2.5 constant A for 7 min and Ponceau staining (0.1% Ponceau S (w/v) in 5% acetic acid) was performed.

The blots were incubated in immunoblotting buffer (TBST: 20 mM Tris-HCl, pH 7.4, 0.15 M NaCl, 0.05% Tween-20) (Bio-Rad) containing 5% (w/v) Skim Milk Powder at room temperature, followed by washing with TBST. Blots were then incubated overnight at 4 °C with either anti-CML antibodies (1:800) in TBST containing 5% milk, or Anti-HNE polyclonal antibody (1:800) or anti-RAGE antibodies (1:400) both in TBST containing 3% milk. The blots were washed three times with TBST, then incubated in Goat Anti-Rabbit IgG, HRP (horseradish peroxidase)-conjugate (Jackson Immune Research) 1:10000 in TBST, in the case of CML, or with Goat anti-rabbit IgG (H + L) IgG (Invitrogen) 1:1000 in TBST containing 3% milk, in the case of HNE and Rabbit Anti-Goat IgG, HRP-conjugate (Sigma) 1:50000 in TBST, in the case of RAGE. After washing the blots with TBST, CML and HNE modified proteins or RAGE were visualized with Immun-Star WesternC Chemiluminescent Kit (BioRad).

After washing with TBST, the blots were blocked and probed in the same manner as stated above. Anti-beta actin, freshly diluted 1:10000 in TBST containing 3% nonfat dry milk, was used as primary antibody and goat anti-mouse IgG1(gamma1) HRP (Invitrogen) was used as the secondary antibody.

All immunoblot images were scanned by ChemiDoc MP (Bio-Rad) and densitometric quantifications were performed using Image Lab software (Bio-Rad). Total CML and HNE were quantified using the intensity of the whole lane, whereas RAGE protein expression was quantified using only the intensity of the stained bands. Beta-actin intensity was used to normalize CML, HNE or RAGE intensity.

### Liver and kidney histology

Liver and renal histology was performed on formalin fixed and paraffin-embedded tissues. Four-micrometer kidney sections were stained with hematoxylin and eosin, PAS (periodic acid-Schiff) and Masson trichrome; liver sections with hematoxylin and eosin and Masson trichrome. Kidneys specimens were evaluated for glomerulosclerosis, tubulointerstitial injury, and interstitial fibrosis, as previously reported [31]. Liver slides were assessed for steatosis, inflammation and fibrosis, using the NAFLD activity score (NAS) [35].

### Data analysis

Data are reported as means ± SEM for three independent experiments performed in duplicate. ANOVA followed by the Newman Keuls multiple comparison test was used to compare the data. A 0.05 level of probability was set as the minimum criterion of significance.

The statistical analysis was performed using the Prism6 software package (Graphpad Software, San Diego, CA, USA).

Principal component analysis (PCA) of plasma levels of glucose tryglycerides, creatinine, insulin, TNF-α, IL-6 and total cholesterol, urine albumin levels, systolic blood pressure, and body weight gain data was performed using R package “pca3d”: Three Dimensional PCA Plots. R package version 0.8. https://CRAN.R-project.org/package=pca3d

## Results

### Experimental model of metabolic syndrome

**Fig. 1:**
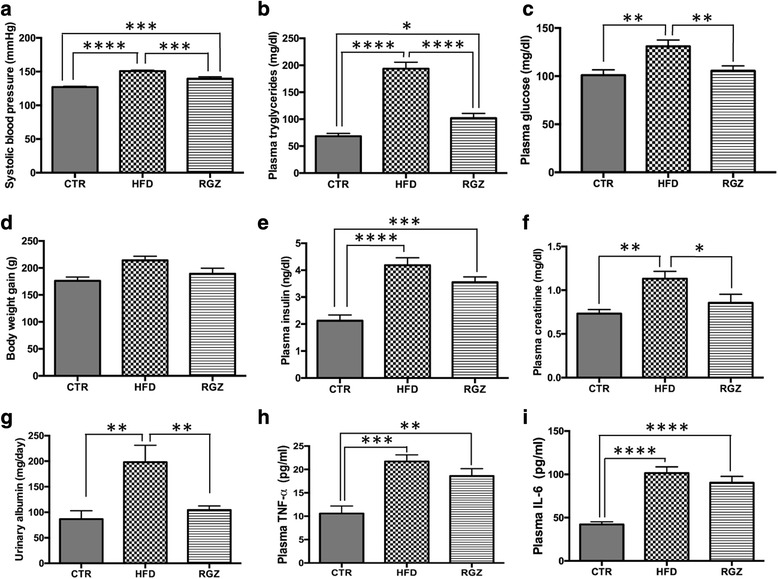
Effect of HFD on established criteria and associated parameters of metabolic syndrome, in the absence and presence of RGZ. Established criteria for metabolic syndrome that were evaluated included: (**a**) systolic blood pressure, (**b**) plasma triglycerides and (**c**) plasma glucose. Several additional parameters often associated with the syndrome were also evaluated: (**d**) body weight, (**e**) plasma insulin, (**f**-**g**) kidney damage and (**h**-**i**) inflammatory cytokine response. Data are mean ± SEM * *P* < 0.05; **P < 0.01; *** *P* < 0.001

In accordance with the currently accepted clinical definition of metabolic syndrome in humans [2], a model of metabolic syndrome (employing a high-fructose diet; HFD) was developed in rats which exhibited the presence of 3 out of the 5 established criteria: (i) hypertension, (ii) high levels of triglycerides, and (iii) increased plasma glucose (Fig. 1a-c, HFD group [middle bars] compared with control group [left bars]). For the remaining two criteria, one (increased body weight) was not significantly changed in this model (Fig. 1d), and the other (decreased HDL cholesterol level) was not measured. Several additional conditions often accompanying the syndrome were also present: insulin resistance (increased plasma insulin) (Fig. 1e), kidney damage (increased plasma creatinine and albuminuria) (Fig. 1f-g) and inflammation (increased plasma levels of TNF-α and interleukin six (IL-6)) (Fig. 1h-i).

No steatosis or fibrosis was observed in the liver in response to the HFD (Additional file 1: Figure S1 A, B), even though several biochemical surrogate indices of impaired liver fat metabolism were present (including increased levels of AST [36, 37], hepatic total lipids [38], hepatic triglycerides [39] and hepatic total cholesterol [40] (Additional file 2: Figure S2)). Similarly, no morphological evidence of renal damage was detected, such as glomerulosclerosis, interstitial inflammatory cell infiltrates, tubular dilatation and/or atrophy and interstitial fibrosis (Additional file 1: Figure S1 D, E). However, albuminuria and increased plasma creatinine levels were found, suggesting some degree of renal injury (Fig. 1f, g).

### PPARγ agonist attenuation of metabolic syndrome

Following three weeks of HFD, addition of RGZ while continuing HFD for the remaining three weeks reduced some physiological parameters of metabolic syndrome in our model: systolic blood pressure, and the levels of both triglycerides and glucose (Fig. 1a-c, HFD group [middle bars] compared with RGZ group [right bars]). RGZ also provided a protective effect from kidney damage as shown by the normalization of plasma creatinine and albumin levels compared with control values (Fig. 1f-g). In contrast, the plasma levels of insulin as well as those of inflammatory markers, were not significantly attenuated by RGZ (Fig. 1e, h, i).

**Fig. 2:**
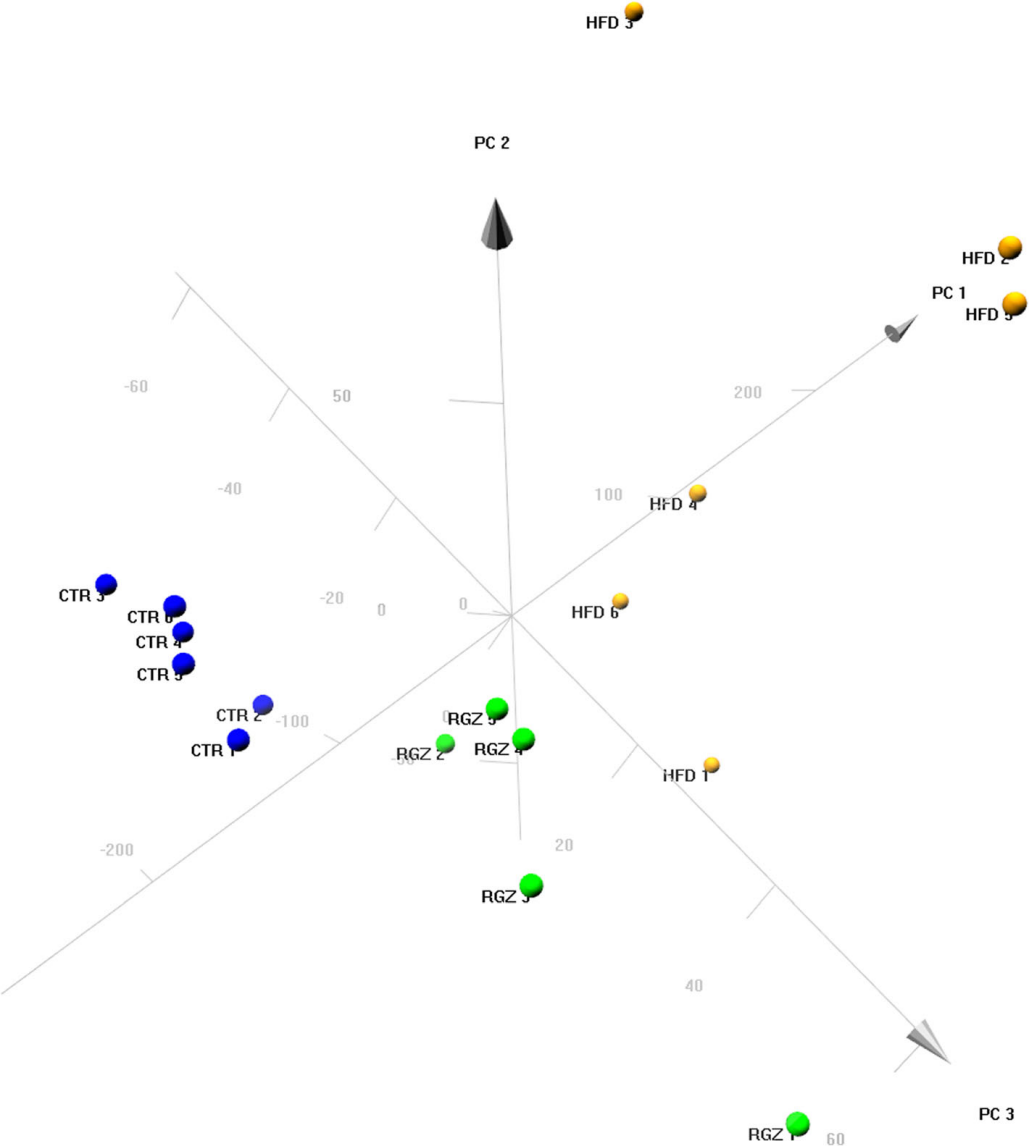
Principal component analysis of results from the three treatment groups (control, HFD, HFD + RGZ) for the multidimentional datasets. Principal component analysis (PCA) was performed for plasma levels of glucose, tryglycerides, creatinine, insulin, TNF-α, IL-6 and total cholesterol, urine albumin levels, systolic blood pressure, and body weight gain data. Results showed that each treatment group clustered separately from the others and that the RGZ group was located between the control and HFD groups

Principle component analysis (PCA) was employed to identify relationship patterns among animals in the three treatment groups (control, HFD, HFD + RGZ) for the multidimentional datasets (i.e., the collection of measured parameters for metabolic syndrome and associated conditions; see Methods for parameter list). The resulting PCA analysis showed that animals from each treatment group were clearly clustered together, and that each group was separated from the other two (Fig. 2). The HFD group was widely separated from the control group, while the RGZ group was located intermediate between the other two groups, in agreement with the overall picture that emerged from the data shown in Fig. 1, namely that HFD induced metabolic syndrome and RGZ significantly attenuated the pathological conditions.

Moreover, the increases in the levels of hepatic triglycerides and hepatic total cholesterol were also prevented by RGZ treatment (Additional file 2: Figure S2D, E), but RGZ did not prevent the increase in levels of AST and hepatic total lipids (Additional file 2: Figure S2A, F).

### Systems biology approach to investigate the AROS axis and lipoxidative protein modifications

We next applied a comprehensive systems biology approach to investigate the complex molecular changes during HFD-induced metabolic syndrome that could be mediated by glycoxidative and lipoxidative protein modifications. We quantified multiple oxidative protein modifications in plasma, urine, kidney and liver, including the levels of AOPP, total AGEs, specific AGEs (CML, CEL) and HNE-protein adducts, as well as RAGE.

### Oxidative damage markers and glycation products in plasma and urine

**Fig. 3:**
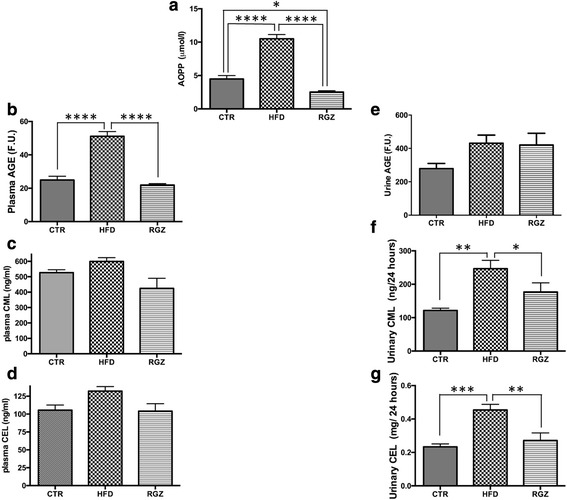
Oxidative damage in plasma and urine. Levels of oxidative stress markers were measured in plasma and urine in response to HFD in the absence and presence of RGZ. **a** plasma AOPP; (**b**) plasma total AGEs; (**c**) plasma CML; (**d**) plasma CEL; (**e**) urine total AGEs; (**f**) urine CML and (**g**) urine CEL. Data are mean ± SEM * *P* < 0.05; **P < 0.01; *** *P* < 0.001

Oxidative damage to proteins in plasma from the HFD group (as detected by AOPP) was significantly increased (>2-fold) and completely prevented by RGZ; in fact, RGZ significantly suppressed AOPP levels below those of control animals (Fig. 3a, HFD group [middle bars] compared with RGZ group [right bars]). HFD also increased the levels of total AGEs in plasma, while the levels of these toxic products were maintained at control levels by RGZ (Fig. 3b). Interestingly, levels of two specific AGEs, CML and CEL, in plasma were not altered by either HFD or RGZ (Fig. 3c, d). In urine, the level of total AGEs was not changed (Fig. 3e), but both CML and CEL exibited > 2-fold increases in response to HFD and a counteracting effect from RGZ (Fig. 3f, g).

### Glycation and RAGE expression in kidney and liver

Kidney and liver tissues lysates were separated into a Triton X-100 soluble fraction (Sup) (representing essentially a total cell lysate) and a Triton X-100 insoluble fraction (Pellet), which enriches for specific detergent resistant membranes (e.g., lipid rafts) [41]. This fractionation scheme was important because the receptor for AGEs (RAGE) is localized in two membrane compartments: (i) the plasma membrane (Triton X-100 soluble “Sup” fraction, in which RAGE signalling is inactive), and (ii) lipid rafts (where active RAGE signalling occurs) [42, 43].

**Fig. 4:**
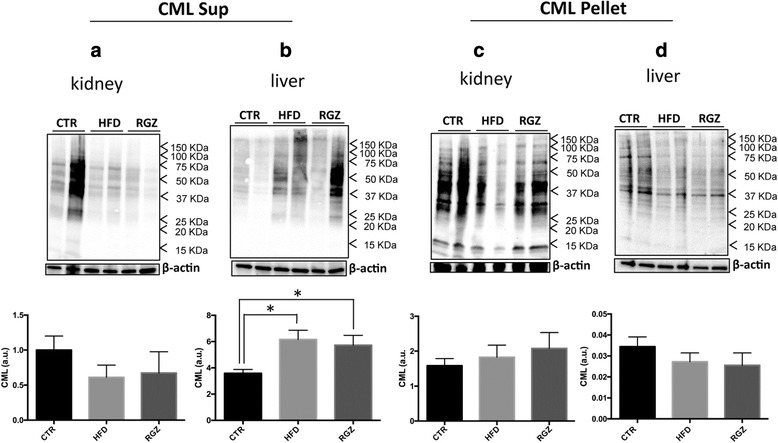
Quantitative immunoblotting of CML adducts in subcellular fractions from kidney and liver. The levels of the specific AGEs CML in response to HFD in the absence and presence of RGZ were quantified in subcellular fractions (Sup and Pellet) from kidney and liver. Representative immunoblots are shown in each panel along with the quantitation. Levels of CML in Sup fractions from (**a**) kidney and (**b**) liver; levels of CML in Pellet fractions from (**c**) kidney and (**d**) liver. Sup = Triton X-100 soluble fraction; Pellet = Triton X-100 insoluble fraction, which enriches for detergent resistant membranes (e.g., lipid rafts). Data are reported as means ± SEM for three independent experiments performed in duplicate. *P < 0.05

First, the level of glycation (CML) was increased almost 2-fold in the Sup fraction of the liver from the HFD group (Fig. 4b, control group [left bars] compared with HFD group [middle bars]). At this stage of the syndrome, we did not detect any other significant change in the levels of CML, including in the Pellet fraction from liver (Fig. 4d) nor in either fraction from kidney (Fig. 4a, c). RGZ had no effect on the levels of CML in either liver or kidney (Fig. 4a-d).

**Fig. 5:**
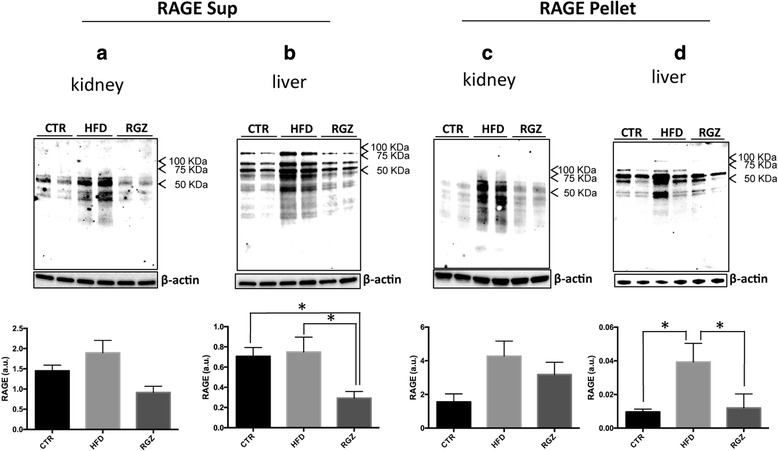
Quantitative immunoblotting of RAGE expression levels in subcellular fractions from kidney and liver. The levels of RAGE expression in response to HFD in the absence and presence of RGZ were quantified in subcellular fractions (Sup and Pellet) from kidney and liver. Representative immunoblots are shown in each panel along with the quantitation. Levels of RAGE in Sup fractions from (**a**) kidney and (**b**) liver; levels of RAGE in Pellet fractions from (**c**) kidney and (**d**) liver. Sup = Triton X-100 soluble fraction; Pellet = Triton X-100 insoluble fraction, which enriches for detergent resistant membranes (e.g., lipid rafts). Data are reported as means ± SEM for three independent experiments performed in duplicate. *P < 0.05

No statistically significant increase in RAGE levels were detected in the kidney in response to HFD, and no significant attenuation was induced by RGZ treatment (Fig. 5a, c). Similarly, no change in levels of inactive RAGE (Sup fraction) in liver resulted from HFD (Fig. 5b). In contrast, HFD induced a dramatic 4-fold increase in levels of active RAGE (Pellet fraction) in liver (Fig. 5d). Furthermore, in liver, RGZ treatment resulted in significantly decreased levels of inactive RAGE (Sup fraction) (to 50% of control levels) and normalization of levels of active RAGE (Pellet fraction) (Fig. 5b, d).

### Lipoxidation in plasma, kidney and liver

**Fig. 6:**
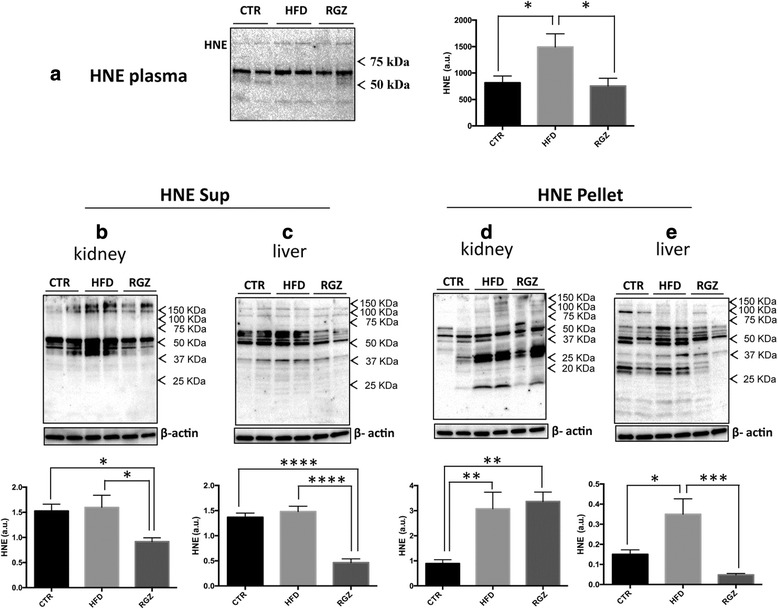
Quantitative immunoblotting of HNE-protein adduct levels in plasma and subcellular fractions from kidney and liver. The levels of HNE-protein adducts formed in response to HFD in the absence and presence of RGZ were quantified in plasma and subcellular fractions (Sup and Pellet) from kidney and liver. Representative immunoblots are shown in each panel along with the quantitation. **a** Levels of plasma HNE; levels of HNE in Sup fractions from (**b**) kidney and (**c**) liver; levels of HNE in Pellet fractions from **d**) kidney and (**e**) liver. Sup = Triton X-100 soluble fraction; Pellet = Triton X-100 insoluble fraction, which enriches for detergent resistant membranes (e.g., lipid rafts). Data are reported as means ± SEM for three independent experiments performed in duplicate. * *P* < 0.05; **P < 0.01; *** *P* < 0.001

The level of HNE-modified proteins in plasma was increased almost 2-fold by HFD treatment, while RGZ completely normalized the level of HNE adducts (Fig. 6a).

In the Sup fraction from both kidney and liver, the level of HNE adducts did not increase in response to HFD, while RGZ treatment resulted in a significant reduction below control levels (1.7- and 3.5-fold reductions for kidney and liver, respectively) (Fig. 6b, c). In marked contrast to results with the Sup fraction (total cell lysate), HFD induced a significant increase in HNE-modified proteins in the Pellet fraction (detergent-resistant membranes/lipid rafts) from both kidney and liver (3.3- and 2.3-fold, respectively) (Fig. 6d, e). While RGZ had no effect on the elevated levels of HNE adducts in the kidney pellet fraction, RGZ treatment normalized the HNE adduct levels in the liver pellet fraction (similar to the result observed for RAGE levels in this fraction (Fig. 5d)).

## Discussion

We employed a rat model for metabolic syndrome that fulfilled the established criteria for this syndrome, exhibiting three out of the five conditions required [2]: increased blood pressure and increased levels of both glucose and triglycerides. In addition, our rat model exhibited other conditions often associated with metabolic syndrome, including increased insulin resistance, kidney dysfunction, and increases in indices of impaired liver fat metabolism (i.e., increased levels of AST, hepatic total lipids, hepatic triglycerides and hepatic total cholesterol). Collectively, these data indicate that our rat model adequately recapitulated the metabolic syndrome.

**Fig. 7:**
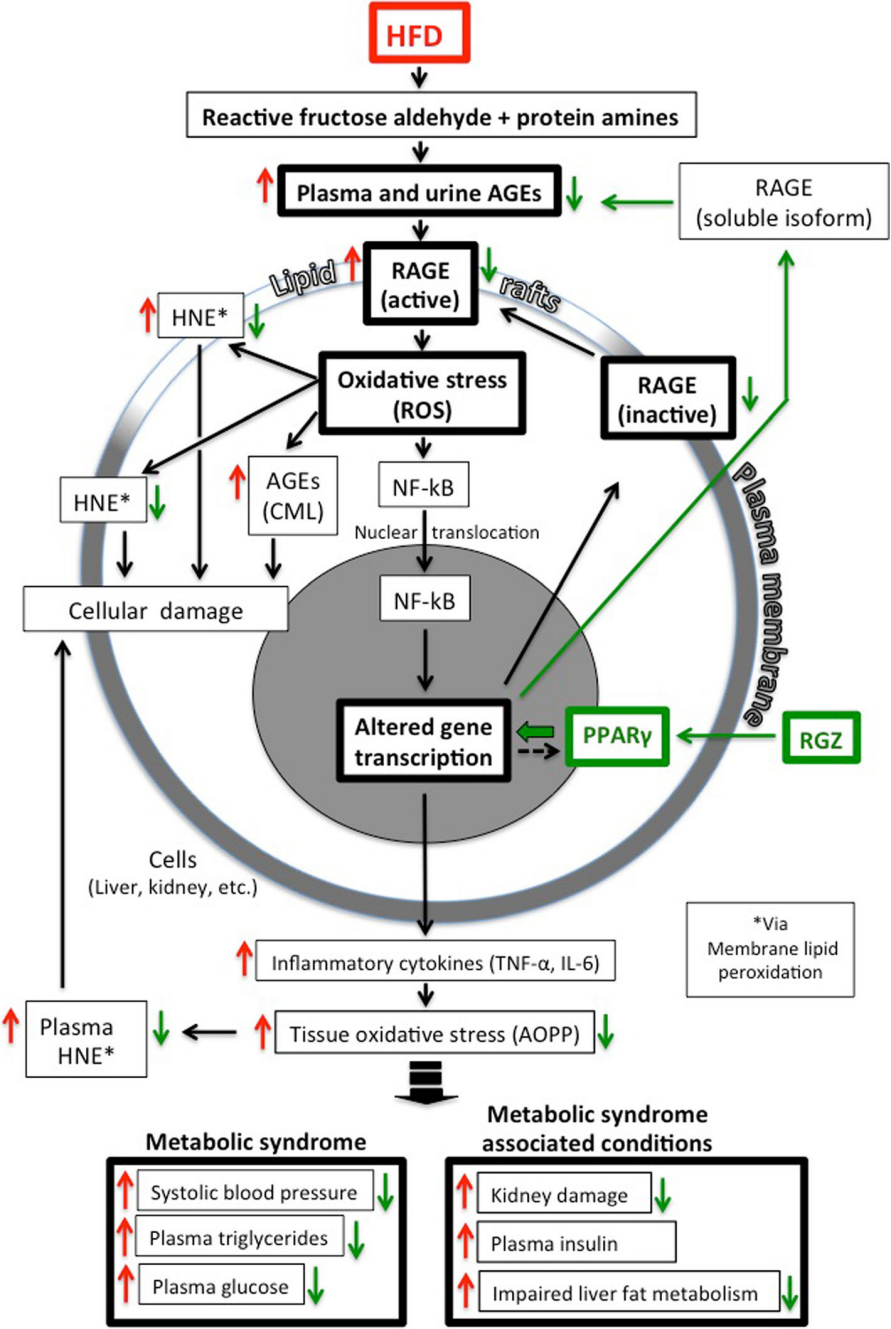
Diagram depicting the AROS axis and summarizing our results. The metabolic syndrome is probably generated through the activation of the AROS axis [8]. Reactive sugar aldehydes form adducts with proteins (AGEs) which then bind to their specific receptor (RAGE, localized in a specialized membrane compartment, the lipid raft), initiating the RAGE signalling cascade. As a result, enzymes generating reactive oxygen species (ROS) are activated, which in turn activates NF-kB translocation from cytosol to nucleus, ultimately altering expression of numerous genes, including upregulation of pro-inflammatory cytokines (TNF-α, IL-6). The resulting inflamation, in turn, is responsible for additional tissue oxidative stress (AOPP production) [49], lipoxidation and HNE production [9–13], all contributing to a further elevation and perpetuation of both molecular/cellular damage and the inflammatory response [50]. Activation of PPARγ binding by RGZ also leads to altered expression of numerous genes, including upregulation of a soluble isoform of RAGE able to bind extracellular AGEs for clearance from the plasma [51], thereby attenuating (at least in part) the AROS axis. Summary of results from this study: (i) upward *red arrows* = increased levels of the indicated measured parameters in response to HFD; (ii) downward *green arrows* = decreased levels of the indicated measured parameters in response to RGZ

A comprehensive systems biology approach was undertaken to examine the AROS axis during development of metabolic syndrome and the response to treatment with a PPARγ agonist (RGZ). A diagram depicting the AROS axis and summarizing our results is shown in Fig. 7. In response to HFD, virtually every measured parameter of the AROS axis increased (upward red arrows). Conversely, activation of PPARγ with RGZ resulted in significant attenuation of nearly every measured parameter of the AROS axis, as well as attenuation of the defining parameters of the metabolic syndrome itself (downward green arrows).

Although many of the results from the current study are consistent with previous reports using various models of metabolic syndrome, this is one of the most comprehensive studies using a single model to characterize both the syndrome and its attenuation by a PPARγ agonist. Despite the overall agreement of results from this study with previous ones, a few discrepancies should be mentioned.

The first issue to mention is body weight gain, which did not occur in our model. Although weight gain has been shown to be an early risk factor in the development of non-alcoholic fatty liver disease (NAFLD) [44], it has been reported that only 60% of metabolic syndrome patients actually gain weight [1]. Thus, this manifestation is not necessarily required for development of metabolic syndrome, even in humans, and also may not occur in some rat models of metabolic syndrome. In a related issue, treatment of diabetic patients with thiazolidinediones has also been reported to result in weight gain [16], but this may also be an effect specific to humans. Similar to our results with RGZ, in numerous animal models with HFD alone or HFD in combination with other diets, the thiazolidinidones did not induce additional weight gain in rats with metabolic syndrome [45–48].

Second, some recent evidence has been presented supporting the hypothesis that steatosis (associated with NAFLD) is a precursor to development of metabolic syndrome, at least in humans [36]. In our rat model, which also exhibited several biochemical indicators of steatosis-associated dyslipidemias (Additional file 2: Figure S2), no histological evidence of frank steatosis was observed (Additional file 1: Figure S1). Collectively, these data could indicate that our model of metabolic syndrome, while exhibiting functional features of abnormal fat metabolism, has not yet reached an advanced stage in which morphologically assessable manifestations have occurred in the liver. Alternatively, steatosis may not be a necessary precursor for metabolic syndrome in rats.

Ultimately, differences in findings among the numerous models employed may be accounted for by the various differences in the individual models themselves, including as examples: (i) differences between humans and rodents, (ii) significantly different diet compositions, (iii) different durations of both diet and drug treatments, and (iv) age of the animals, among others.

## Conclusions

Despite extensive efforts to completely understand the metabolic syndrome, its exact pathogenic mechanism at the molecular level remains incompletely defined, even in the HFD rat model [4]. There is a strong consensus that the AROS axis (i) plays a central role in induction of the metabolic syndrome and (ii) is a target for the counteracting effects of PPARγ agonists. In this study, we applied a systems biology approach for comprehensive evaluation of the multiple molecular modifications taking place via stimulation of the AROS axis by HFD, both without and with agonist potentiation of PPARγ activity by RGZ. The results provide a more complete picture of the landscape of the AROS axis, and the study serves as a proof-of-concept approach for evaluating new drug candidates. Further characterization and identification of the glycated and lipoxidated proteins using this strategy should provide deeper insights into their functional role in the complex molecular mechanisms involved in metabolic syndrome.

## Additional files

Additional file 1: Figure S1. Representative images of liver and kidney histological sections. No significant difference for steatosis or fibrosis was observed in liver (upper row) between the groups. A) CTR, (B) HFD, (C) RGZ; no morphological evidence of damage, such as glomerulosclerosis, interstitial inflammatory cell infiltrates, tubular dilatation and/or atrophy and interstitial fibrosis, was detected in kidney (lower row): (D) CTR, (E) HFD, (F) RGZ. Images were acquired with NanoZoomer-XR C12000 series, scale bars = 250 μm. (JPG 82 kb)
Additional file 2: Figure S2. Biochemical surrogate indices of impaired liver fat metabolism. In response to HFD, increased levels of surrogate indices of steatosis/dyslipidemia were observed, including: A) aspartate aminotransferase (AST), D) hepatic triglycerides, E) hepatic total cholesterol and F) hepatic total lipids, but not B) alanine transaminase (ALT) or C) plasma total cholesterol. Moreover, the increase in the levels of hepatic triglycerides (D) and hepatic total cholesterol (E) were significantly reduced by RGZ treatment, but RGZ did not significantly affect the increase in the levels of AST (A) or hepatic total lipids (F). Data are mean ± SEM * *P* < 0.05; ***P* < 0.01; *** *P* < 0.001. (JPG 45 kb)

## Abbreviations

AGEs: Advanced glycation end products
ALEs: Advanced lipoxidation end products
AOPP: Advanced oxidation protein products
CEL: Carboxyethyl lysine
CML: Carboxymethyl lysine
CTR: Control
HFD: High fructose diet
HNE: 4-hydroxynonenal
NO: Nitric oxide
RAGE: Receptor for advanced glycation end products
RGZ: Rosiglitazone
