# Supplementary figures and images for: Regulatory landscape of AGE-RAGE-oxidative stress axis and its modulation by PPARγ activation in high fructose diet-induced metabolic syndrome
Source: Nutr Metab (Lond). 2017 Jan 13;14:5. doi: 10.1186/s12986-016-0149-z (PMC5237238; doi:10.1186/s12986-016-0149-z)

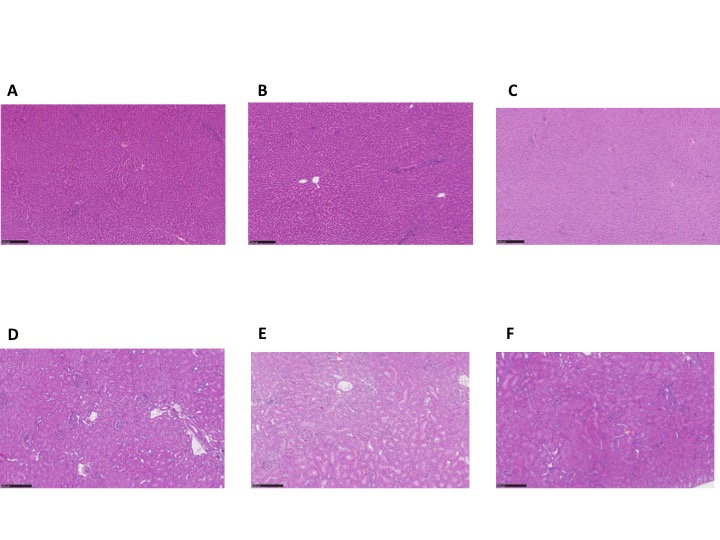

Supplement: Additional file 1: Figure S1. — Representative images of liver and kidney histological sections. No significant difference for steatosis or fibrosis was observed in liver (upper row) between the groups. A) CTR, (B) HFD, (C) RGZ; no morphological evidence of damage, such as glomerulosclerosis, interstitial inflammatory cell infiltrates, tubular dilatation and/or atrophy and interstitial fibrosis, was detected in kidney (lower row): (D) CTR, (E) HFD, (F) RGZ. Images were acquired with NanoZoomer-XR C12000 series, scale bars = 250 μm. (JPG 82 kb) [file 12986_2016_149_MOESM1_ESM.jpg]

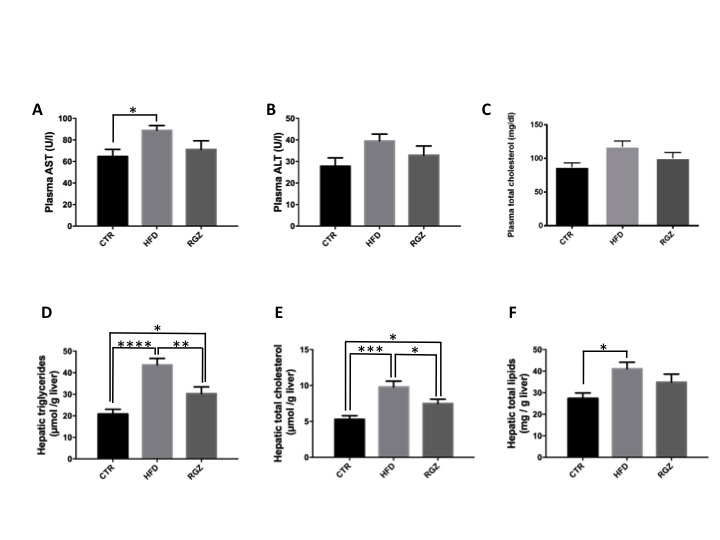

Supplement: Additional file 2: Figure S2. — Biochemical surrogate indices of impaired liver fat metabolism. In response to HFD, increased levels of surrogate indices of steatosis/dyslipidemia were observed, including: A) aspartate aminotransferase (AST), D) hepatic triglycerides, E) hepatic total cholesterol and F) hepatic total lipids, but not B) alanine transaminase (ALT) or C) plasma total cholesterol. Moreover, the increase in the levels of hepatic triglycerides (D) and hepatic total cholesterol (E) were significantly reduced by RGZ treatment, but RGZ did not significantly affect the increase in the levels of AST (A) or hepatic total lipids (F). Data are mean ± SEM * P < 0.05; **P < 0.01; *** P < 0.001. (JPG 45 kb) [file 12986_2016_149_MOESM2_ESM.jpg]
